# Immunomodulatory effects of synthetic antimicrobial peptides on LPS-induced inflammatory responses in THP-1 macrophages

**DOI:** 10.3389/fimmu.2026.1829304

**Published:** 2026-06-10

**Authors:** Ilayda Akbulut, Ziyun Zhang, Tracy Hussell, Jeremy P. Derrick, Jian Ren Lu

**Affiliations:** 1 Biological Physics Laboratory, Department of Physics and Astronomy, School of Natural Sciences, Faculty of Science and Engineering, The University of Manchester, Manchester, United Kingdom; 2 Lydia Becker Institute of Immunology and Inflammation Division of Infection, Immunity & Respiratory Medicine, School of Biological Sciences, Faculty of Biology, Medicine and Health, The University of Manchester, Manchester, United Kingdom

**Keywords:** antimicrobial peptides, inflammatory kinetics, innate immune modulation, lipopolysaccharide neutralization, macrophage polarization, THP-1-derived macrophages

## Abstract

Macrophage polarization is critical for maintaining health and for recognizing and eliminating a diverse array of antigens. These disparate roles require distinct macrophage responses, and the induction of inappropriate macrophage responses is intimately associated with disease. It is therefore important to understand how macrophages respond to new biomedicines and to harness those with immune modulatory properties for therapeutic use. This study defines the effects of rationally designed antimicrobial peptides (AMPs) on macrophage polarization and function, compared with natural host-defense peptides (natural AMP LL-37 and antibiotic polymyxin B). Using an *in vitro* model system, our results show that the designed AMPs suppress pro-inflammatory cytokine production while enhancing anti-inflammatory responses, with potency comparable to LL-37, and mitigate the impact of LPS-induced inflammation. Furthermore, AMPs influence key molecular pathways, such as IRF3/IRF4 and PPAR-γ, favoring M2 polarization. These results highlight the amphiphilic, sequence-engineered design of the peptides, which enables controlled membrane interaction, low cytotoxicity, and structural adaptability, properties central to next-generation immune-active biomaterials. By coupling antimicrobial function with immune reprogramming, these synthetic AMPs act as dual-function agents capable of alleviating inflammatory conditions such as cytokine storm and promoting reparative macrophage phenotypes. This work identifies a new design framework for bioinspired, immunomodulatory peptide materials with potential applications in infection control, inflammatory disease management, and wound healing.

## 1 Introduction

Increasing resistance to antibiotics necessitates the development of new and innovative therapeutic strategies. Multidrug resistance is particularly devastating and could set medicine back to the preantibiotic era (1–3). Among alternative approaches, antimicrobial peptides (AMPs) have gained significant attention as bioactive molecules that can overcome conventional resistance mechanisms through distinct, non-enzymatic modes of action. Current antibiotics primarily function by targeting specific receptors and impairing their functions through interference with normal enzymatic, metabolic, and genetic processes (4–6). In contrast, AMPs kill bacteria predominantly by disrupting membrane integrity. Owing to this rapid, physical mechanism of membrane disruption, bacteria are less able to adapt (7), thereby reducing the likelihood of AMP resistance development (8).

As bioactive peptides, AMPs exhibit remarkable antibacterial, antiviral, antifungal, antiparasitic, and anticancer properties and constitute an essential first line of defense in the human innate immune system (9). Most AMPs are short (12–50 amino acids), amphiphilic, and carry two to nine positive charges (6). The advantages of AMPs include their broad-spectrum antibacterial activity, great stability, superior solubility, and structural diversity across species. Benefiting from their distinct membrane-lytic mode of action, AMPs are also effective against clinically isolated antibioticresistant strains without readily inducing bacterial resistance (10). Human cathelicidin LL-37, the only naturally occurring human cathelicidin, displays broad activity against bacteria, fungi, and viruses while also promoting wound healing and immune modulation (11–13). However, natural AMPs often face challenges in clinical development due to high production costs, instability against proteolysis, cytotoxicity, and potential off-target side effects (2, 3). To overcome these limitations, numerous biomimetic and rationally designed AMPs have been developed through sequence optimization, including truncation and amino acid substitution. These synthetic AMPs are costeffective, potent, and biocompatible with reduced toxicity. G_3_ is a novel designed AMP consisting of three repeats of the amphipathic *IIKK* (isoleucine-isoleucine-lysine-lysine) motif (14). Its structural analogues, C_8_G_2_ and C_12_G_2_, contain two *IIKK* repeats and are modified at the N-terminus with caprylic (C_8_) and lauric (C_12_) acid chains, respectively (7, 15). The molecular structures of G_3_, C_8_G_2_ and C_12_G_2_ are shown in **Supplementary Figure 1**.

Beyond their antibacterial activity, AMPs play important roles in regulating immune responses, facilitating pathogen clearance, mitigating the detrimental effects of excessive inflammation, and reshaping innate immune cell signaling and metabolism (16–18). Furthermore, AMPs also promote wound healing and help regulate the transition to adaptive immunity (5, 19, 20). For instance, by directly binding lipopolysaccharide (LPS) or preventing its interaction with host cell receptors, LL-37 neutralizes the inflammatory potential of LPS and disrupts signaling cascades initiated by Toll-like receptor (TLR) ligands (21). Following tissue injury, immune regulation is essential to prevent bystander tissue damage that may result in long-term complications with the risk of increased susceptibility to bacterial infection (22–24). Accordingly, the design of innovative AMPs with combined antibacterial and anti-inflammatory properties is critical for effective wound healing. Synthetic peptides capable of suppressing pro-inflammatory responses while improving wound healing, immune cell survival, and chemotaxis have been reported (23–25). Under wound-mimicking conditions, AMPs can bind and neutralize LPS, thereby preventing excessive pro-inflammatory cytokine production by macrophages. In addition, AMPs can modulate immune responses to limit inflammation during tissue repair while supporting immune cell recruitment for efficient bacterial eradication. Although the physicochemical characteristics and their modes of actions of AMPs have been thoroughly examined, relatively little research has been undertaken to unravel how synthetic AMPs modulate immunoregulatory pathways (19–21).

Macrophages are an important component of the innate immune system and are either developed embryonically from the yolk sac or derived from bone marrow monocytes (26–28). Macrophages exhibit substantial heterogeneity and plasticity, which are regulated by subtle alterations in the environmental cues (29). More recent concepts in innate immune biology further highlight that macrophage responses are not solely defined by acute polarization states, but also by metabolic and epigenetic reprogramming processes that shape subsequent inflammatory responsiveness, including trained immunity and immune tolerance (30–32). These adaptive innate programs are closely linked to shifts in glycolysis, oxidative phosphorylation, and lipid metabolism that influence macrophage inflammatory phenotypes (32). Macrophage plasticity and heterogeneity are driven by tissue specific signals that are dependent on the needs of the tissue at that time (33). Macrophages perform diverse functions, including phagocytosis of pathogens (34), eliminating waste materials and dead cells (35), producing cell activating cytokines and chemokines (36), tissue remodeling (37), and regulation of other immune cell populations (38–40). These functions require precise macrophage reprogramming, with the two extremes commonly defined as M1 (classically activated or pro-inflammatory) and M2 (alternatively activated or anti-inflammatory) phenotypes (41), although macrophage states are now recognized to exist along a dynamic continuum rather than as fixed binary subsets (30, 32). M1 macrophages are potent effector cells that eliminate microorganisms and produce copious proinflammatory cytokines and specific chemokines, whereas M2 macrophages primarily contribute to the resolution of inflammation, debris clearance, angiogenesis, tissue remodeling, and wound healing and repair (42–44). As major cytokine producers at sites of inflammation, macrophages represent an ideal model for investigating inflammatory processes and possible therapeutic interventions.

**Figure 1:**
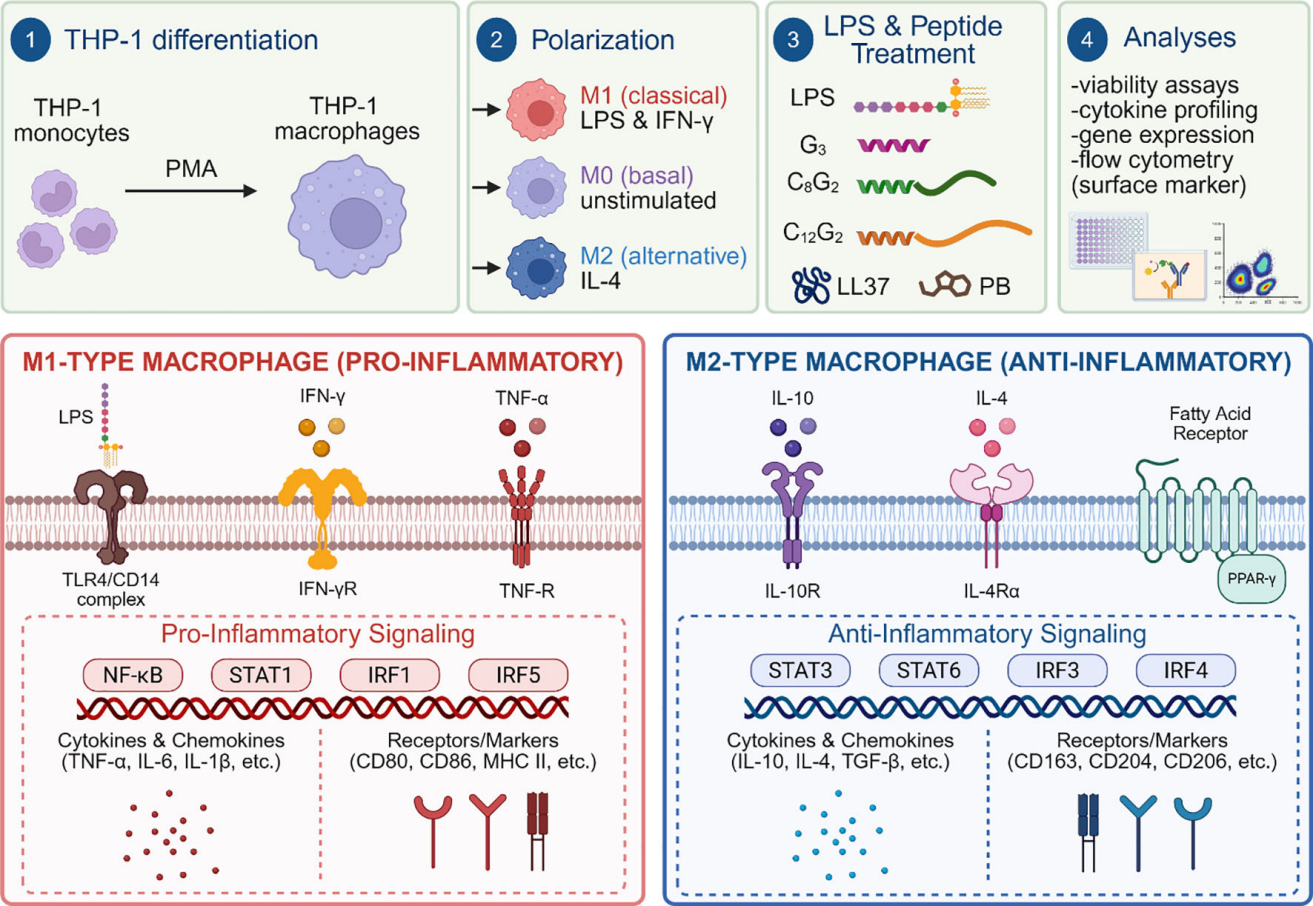
Experimental workflow for THP-1 macrophage differentiation, polarization, and peptide treatment, and analysis. THP-1 monocytes were differentiated into M0 macrophages using PMA, then polarized into M1 (IFN-γ & LPS) or M2 (IL-4) phenotypes. Cells were treated with antimicrobial peptides (G_3_, C_8_G_2_, C_12_G_2_, LL-37), and antibiotic PB, with and without LPS. Experimental readouts included viability assays, cytokine profiling, gene expression analysis, and flow cytometry. Lower panels summarize representative M1-associated pro-inflammatory pathways and M2-associated anti-inflammatory pathways, cytokines, and surface markers evaluated in this study.

The aim of this study was to establish a robust *in vitro* macrophage model to systematically evaluate how rationally designed AMPs influence macrophage polarization, activation, and inflammatory responses. Using the human monocytic cell line THP-1, we first optimized differentiation and polarization protocols to generate well-defined M0, M1, and M2 macrophage phenotypes. We then assessed the immunomodulatory properties and cytotoxicity of the synthetic AMPs G_3_, C_8_G_2_, and C_12_G_2_ in comparison with the natural AMP LL-37 and the antibiotic polymyxin B (PB) (43). By integrating analyses of cell viability, inflammatory cytokine kinetics, surface marker expression, and gene expression profiles, we investigated how these peptides modulate LPS-induced macrophage activation. This approach addresses a critical gap in understanding how sequence-engineered synthetic AMPs regulate macrophage polarization, inflammatory signaling, and potentially broader macrophage reprogramming pathways linked to immunometabolism, beyond their established antimicrobial activity. The overall experimental workflow used in this study is summarized in **Figure 1**.

## 2 Materials and methods

### 2.1 Reagents and experimental design

RPMI-1640 medium and 3-(4, 5-dimethlythiazol-2-yl)-2, 5-diphenyltetrazolium bromide (MTT) were purchased from Sigma-Aldrich, St. Louis, MO, USA. Lipopolysaccharide (LPS) from *Escherichia coli* strain ATCC 25922 was obtained from ATCC, Manassas, VA, USA, and prepared as a 1 mg/mL stock solution in endotoxin-free water, aliquoted, and stored at −20 °C. Recombinant human IFN-γ and IL-4 were purchased from PeproTech, Rocky Hill, NJ, USA. Fetal calf serum from (FCS; Gibco, Thermo Fisher Scientific, Waltham, MA, USA), phosphate-buffered saline (PBS; pH 7.4), penicillin/streptomycin (P/S) and dimethyl sulfoxide (DMSO) were obtained from Sigma-Aldrich, St. Louis, MO, USA. Phorbol 12-myristate-13 acetate (PMA; molecular weight: 616 g/mol; SigmaAldrich, St. Louis, MO, USA) was prepared as a 2 mg/mL stock solution in DMSO, aliquoted and stored at −20 °C. Working solutions were freshly prepared in culture medium to a final concentration of 100 ng/mL.

ELISA kits for TNF-α, IL-4, IL-6, and IL-10 were purchased from R&D Systems, Minneapolis, MN, USA. Synthetic peptides G_3_, C_8_G_2_, and C_12_G_2_ (≥98% purity, verified by RP-HPLC and MS) were obtained from GL Biochem Ltd., Shanghai, China. LL-37 was purchased from QYAOBIO, Shanghai, China and polymyxin B sulphate was obtained from Sigma Aldrich, St. Louis, MO, USA. Peptides and antibiotic were dissolved in PBS or endotoxin-free water to prepare 2 mM stock solutions and diluted to the desired working concentration.

RNA isolation reagents were obtained from the RNeasy Mini Kit (Qiagen, Hilden, Germany). cDNA synthesis was performed using the High-Capacity RNA-to-cDNA Kit (Applied Biosystems, Thermo Fisher Scientific, Waltham, MA, USA), and quantitative PCR was carried out using PowerUp™ SYBR™ Green Master Mix (Applied Biosystems, Thermo Fisher Scientific, Waltham, MA, USA). All primers were purchased from Sigma-Aldrich, St. Louis, MO, USA, and the sequences used are listed in **Supplementary Table 1**. Human FcR Blocking Reagent was obtained from Miltenyi Biotec, Bergisch Gladbach, Germany. Human monoclonal antibodies used for flow cytometry are listed in **Table 1**. Live/dead stain (Zombie L/D 355 nm) obtained from BD Biosciences, San Jose, CA, USA.

**Table 1:** List of antibodies used for flow cytometry analysis.

*Cell Surface Marker*	*Function*	*Dilution*	*Fluorophore*	*Clone Number*	*Manufacturer*
CD11b	Cell adhesion and migration	1:200	BV711	M1/70	BioLegend
CD14	LPS receptor	1:200	PE/Cy7	HCD14	BioLegend
CD68	Macrophage marker	1:200	APC/Cy7	Y1/82A	BioLegend
CD80	Activate T cells	1:200	PE/Dazzle 594	2D10	BioLegend
CD86	Costimulatory signaling to promote T-cell activation	1:200	BV650	IT2.2	BioLegend
MHCII (HLA-DR)	Present antigens	1:200	PE/Cy5	L243	BioLegend
CD163	Scavenger receptor	1:200	FITC	GHI/61	BioLegend
CD204	Scavenger receptor-A	1:200	APC	7C9C20	BioLegend
CD206 (MRC1)	Mannose receptor	1:200	PE/Cy7	15-2	BioLegend

### 2.2 Cell culture

The human monocytic THP-1 cell line (ATCC TIB-202™; Manassas, VA, USA) was cultured in RPMI-1640 medium supplemented with 10% heat-inactivated FCS and 1% P/S at 37 °C in a humidified incubator with 5% CO_2_. Cells were maintained at a density between 1 x 10^5^ and 8 x 10^5^ cells/mL and used between passages 3 and 15.

### 2.3 Differentiation of THP-1 monocytes into macrophages

THP-1 monocytes were differentiated into macrophage-like cells by incubation with 100 ng/mL PMA for 48 h (44–46). Following PMA treatment, cells were washed and allowed to rest in PMAfree complete RPMI-1640 medium for an additional 24 h. Differentiation was confirmed by morphological changes, increased adherence, and upregulation of macrophage surface markers CD11b and CD14, as assessed by flow cytometry (28, 48). Cell viability and membrane integrity during differentiation were evaluated using MTT and LDH assays.

### 2.4 Macrophage polarization

Differentiated THP-1 macrophages were defined as naïve macrophages (M0). To generate classically activated macrophages (M1), M0 cells were stimulated with IFN-γ (20 ng/mL) and LPS (1 µg/mL) for 24 h (29, 35). Alternatively activated macrophages (M2) were obtained by treating M0 cells with IL-4 (20 ng/mL) for 24 h (29, 38). Cells treated with PMA alone served as M0 controls. In addition, functional differentiation and polarization of THP-1 derived macrophages into M0, M1, and M2 states were assessed using cytokine profiling and surface marker analysis.

### 2.5 Peptide and LPS treatments

M0 macrophages were treated with LPS (1, 2.5, and 5 μg/mL), antimicrobial peptides (G_3_, C_8_G_2_, C_12_G_2_, LL-37), or polymyxin B at concentrations ranging from 1 to 100 µM. For co-treatment experiments, LPS and peptides or antibiotics were pre-incubated together at 37 °C for 15 min prior to addition to cells. Stimulation periods ranged from 1 h to 48 h depending on the downstream assay. Minimum inhibitory concentration (MIC) values against *E. coli* ATCC 25922 were used for comparative analyses. MIC values for G_3_, C_8_G_2_, C_12_G_2_, LL-37, polymyxin B sulphate are 8, 13, 32, 4, 4 μM respectively.

### 2.6 Cell viability and cytotoxicity assays

Cell viability was assessed using the MTT assay. Differentiated THP-1 macrophages were seeded 2 x 10^4^ cells/well in 96-well plates and treated as indicated. Following incubation, MTT reagent (5 mg/mL) was added and incubated for 2 h. Formazan crystals were solubilized in DMSO, and absorbance was measured at 570 nm using TECAN infinite M200 PRO microplate reader. Untreated controls were defined as 100% viability.

Cytotoxicity was evaluated using a lactate dehydrogenase (LDH) release assay (CytoTox 96^®^ NonRadioactive Cytotoxicity Assay, Promega, Madison, WI, USA) according to the manufacturer’s instructions. LDH activity was measured at 490 nm and expressed as a percentage of maximal release.

### 2.7 Cytokine measurement by ELISA

Cell culture supernatants were collected at specified time points (1–48 h) and stored at −80 °C until analysis. TNF-α, IL-6, IL-4, and IL-10 concentrations were quantified using enzyme-linked immunosorbent assay (ELISA) kits following the manufacturer’s protocols. Absorbance was measured at 450 nm with a reference wavelength of 570 nm. Cytokine concentrations were calculated from standard curves and expressed as pg/mL. All samples were analyzed in biological triplicate.

### 2.8 RNA isolation and RT-qPCR

Total RNA was isolated using the RNeasy Mini Kit according to the manufacturer’s instructions.

RNA purity and concentration were assessed using NanoDrop spectrophotometer (Thermo Fisher Scientific, Waltham, MA, USA), and samples with A260/A280 ratios ≥ 1.8 were used for downstream analysis. cDNA synthesis was performed using 200 ng RNA per reaction.

Quantitative RT-qPCR was performed using PowerUp™ SYBER™ Green Master Mix on a QuantStudio 12K Flex Real-Time PCR System (Applied Biosystems, Thermo Fisher Scientific, Waltham, MA, USA). Gene expression was normalized to the housekeeping gene *B2M* and calculated using the 2^-ΔCt^ method. Results were expressed as fold change relative to M0 control cells. All samples were prepared in biological triplicates. Flow Cytometry.

Cells were stained with Live/Dead Zombie dye, blocked with human Fc receptor blocking reagent, labeled with fluorochrome-conjugated antibodies against macrophage surface markers (**Table 1**). Data was acquired on a BD LSRFortessa™ X20 flow cytometer (BD Biosciences, San Jose, CA, USA) and analyzed using FlowJo software (version 10). Single, live cells were gated based on forward and side scatter properties, and marker expression was quantified as mean fluorescence intensity (MFI) and percentage of positive cells.

### 2.9 Statistical analysis

All data presented as mean ± standard deviation (SD) unless otherwise stated. Statistical analyses were performed using GraphPad Prism (version 9 or later). One-way or two-way ANOVA followed by appropriate *post-hoc* tests (Tukey’s and Dunnett’s) were used as indicated in the figure legends. A *p*-value < 0.05 was considered statistically significant.

## 3 Results

### 3.1 Optimization and validation of THP-1 macrophage differentiation and polarization

THP-1 monocytes were differentiated into macrophage-like cells using an optimized PMA-based protocol (45–47). Upon PMA treatment, THP-1 cells underwent the expected morphological transition from non-adherent, round cells to adherent macrophage-like cell clusters (**Supplementary Figure 2A**). Cell viability, assessed by MTT assay, was not significantly altered compared with medium controls, and LDH release remained low, indicating preserved membrane integrity during differentiation (**Supplementary Figure 2B**). Differentiation was further confirmed by increased surface expression of the macrophage markers CD11b and CD14, as determined by flow cytometry (**Supplementary Figure 2C**). These findings demonstrate effective and reproducible differentiation of THP-1 monocytes into macrophage-like cells.

**Figure 2:**
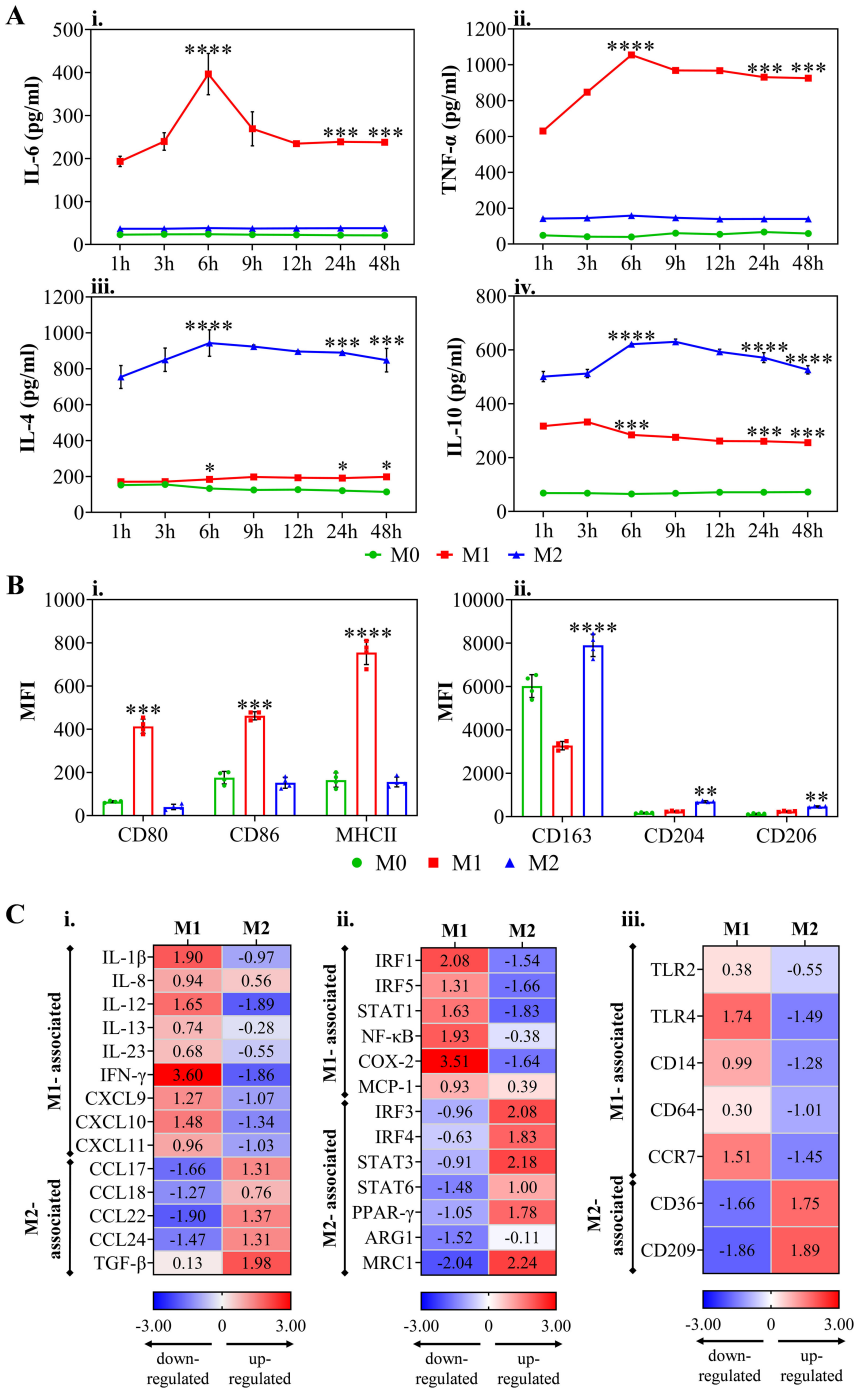
Cytokine, surface marker, and transcriptional validation of THP-1 macrophage polarization **(A)** Cytokine secretion profiles determined by ELISA at 1–48 h following polarization into M0, M1(IFN-γ + LPS), or M2 (IL-4) macrophages: **(i)** IL-6, **(ii)** TNF-α, **(iii)** IL-4, and **(iv)** IL-10 (pg/mL) **(B)** Flow cytometric quantification of macrophage surface markers expressed as mean fluorescence intensity (MFI): **(i)** M1-associated markers (CD80, CD86, and MHC class II) and **(ii)** M2-associated markers (CD163, CD204, and CD206). **(C)** RT-qPCR heatmaps showing log_2_ fold changes in M1- and M2-associated gene expression: **(i)** cytokines and chemokines, **(ii)** transcription factors and effector molecules, and **(iii)** surface markers. Blue indicates downregulation and red indicates upregulation (range: –3.00 to +3.00). Data are presented as mean ± SD (n = 3). Statistical analysis was performed using one-way ANOVA with Tukey’s *post-hoc* test (*p < 0.05; **p < 0.01; ***p < 0.001; ****p < 0.0001; ns, not significant).

To validate macrophage polarization, PMA-differentiated macrophages (M0) were stimulated with IFN-γ and LPS to induce an M1 phenotype or with IL-4 to induce an M2 phenotype. Cell viability and LDH release did not differ significantly among M0, M1, and M2 macrophages (**Supplementary Figure 3A**). Cytokine profiling revealed that M1 macrophages secreted high levels of TNF-α and IL-6, whereas M2 macrophages exhibited elevated IL-4 and IL-10 secretion over 48 h time course (**Figure 2A**). Undifferentiated M0 macrophages showed minimal cytokine release.

Flow cytometric analysis confirmed polarization-specific surface marker expressions. M1 macrophages displayed increased expressions of CD80, CD86, and MHC class II, while M2 macrophages exhibited elevated levels of CD163, CD204, and CD206 (**Figure 2B**). Consistent with these findings, RT-qPCR analysis showed increased expressions of pro-inflammatory cytokines, chemokines, and transcription factors (including *IL-1β*, *IL-8*, *CXCL9-11*, *IRF5*, *STAT1*, and *NF-κB*) in M1 macrophages, whereas M2 macrophages demonstrated higher expressions of antiinflammatory and tissue-repair associated genes, including, *IL-10, TGF-β, IRF3, IRF4, STAT3, STAT6*, and *PPAR-γ* (**Figure 2C**). Together, these data confirm robust and divergent polarization of THP-1 derived macrophages.

### 3.2 LPS induces dose- and time-dependent inflammatory activation in THP-1-derived macrophages

To establish an inflammatory baseline, differentiated THP-1 macrophages were stimulated with increasing concentrations of LPS (1, 2.5, or 5 μg/mL) for up to 48 h. Cell viability analysis showed that LPS concentrations up to 2.5 μg/mL did not significantly affect cell viability, whereas exposure to 5 μg/mL LPS resulted in reduced viability and increased LDH release, indicating cytotoxicity at higher doses (**Supplementary Figure 4A**).

LPS stimulation induced a pronounced pro-inflammatory response. TNF-α and IL-6 secretion increased rapidly, peaking at early time points and remaining elevated over 48 h (**Supplementary Figure 4B**). In contrast, anti-inflammatory cytokines IL-4 and IL-10 remained at baseline levels following LPS stimulation alone. At the highest LPS concentration, cytokine production declined at later time points, consistent with reduced cell viability. These results establish a dose- and time-dependent inflammatory activation profile, mimicked bacterial infection-induced activation in THP-1-derived macrophages and define experimental conditions suitable for evaluating peptide-mediated modulation of LPS-induced inflammation.

### 3.3 Designed AMPs exhibit low cytotoxicity and undergo membrane-associated structural transitions

The cytotoxicity of the designed antimicrobial peptides G_3_, C_8_G_2_, and C_12_G_2_ was evaluated in comparison with the natural AMP LL-37 and polymyxin B. Across a broad concentration range, all peptides exhibited low cytotoxicity toward THP-1-derived macrophages, with minimal reductions in cell viability and limited LDH release (**Supplementary Figure 5**). The low cytotoxicity is overall consistent with previous observations (53). At higher peptide concentrations, modest increases in cytotoxicity were observed, particularly for G_3_ and PB, although these effects remained relatively small and occurred above the minimum inhibitory concentrations (MICs).

The physicochemical properties of the peptides are summarized in **Supplementary Table 2**. All peptides are cationic and amphiphilic, enabling interactions with lipid membranes and LPS. Circular dichroism spectroscopy was used to assess peptide secondary structure in aqueous buffer (PBS or H_2_O) and in SDS micelles as a membrane-mimicking environment. In aqueous conditions, all peptides exhibited predominantly random-coil confirmations, characterized by shallow negative ellipticity near 200 nm (**Supplementary Figure 6**). In contrast, G_3_, C_8_G_2_, C_12_G_2_ and LL-37 adopted pronounced α-helical structures in SDS, with characteristic double minima at approximately 208 and 222 nm. PB exhibited partial structural ordering but did not fully adopt a classical α-helical confirmation. These structural transitions are summarized in **Supplementary Table 3** and indicate environment-dependent confirmational adaptability of the peptides.

### 3.4 AMPs attenuate LPS-induced pro-inflammatory cytokine release

**Figure 3:**
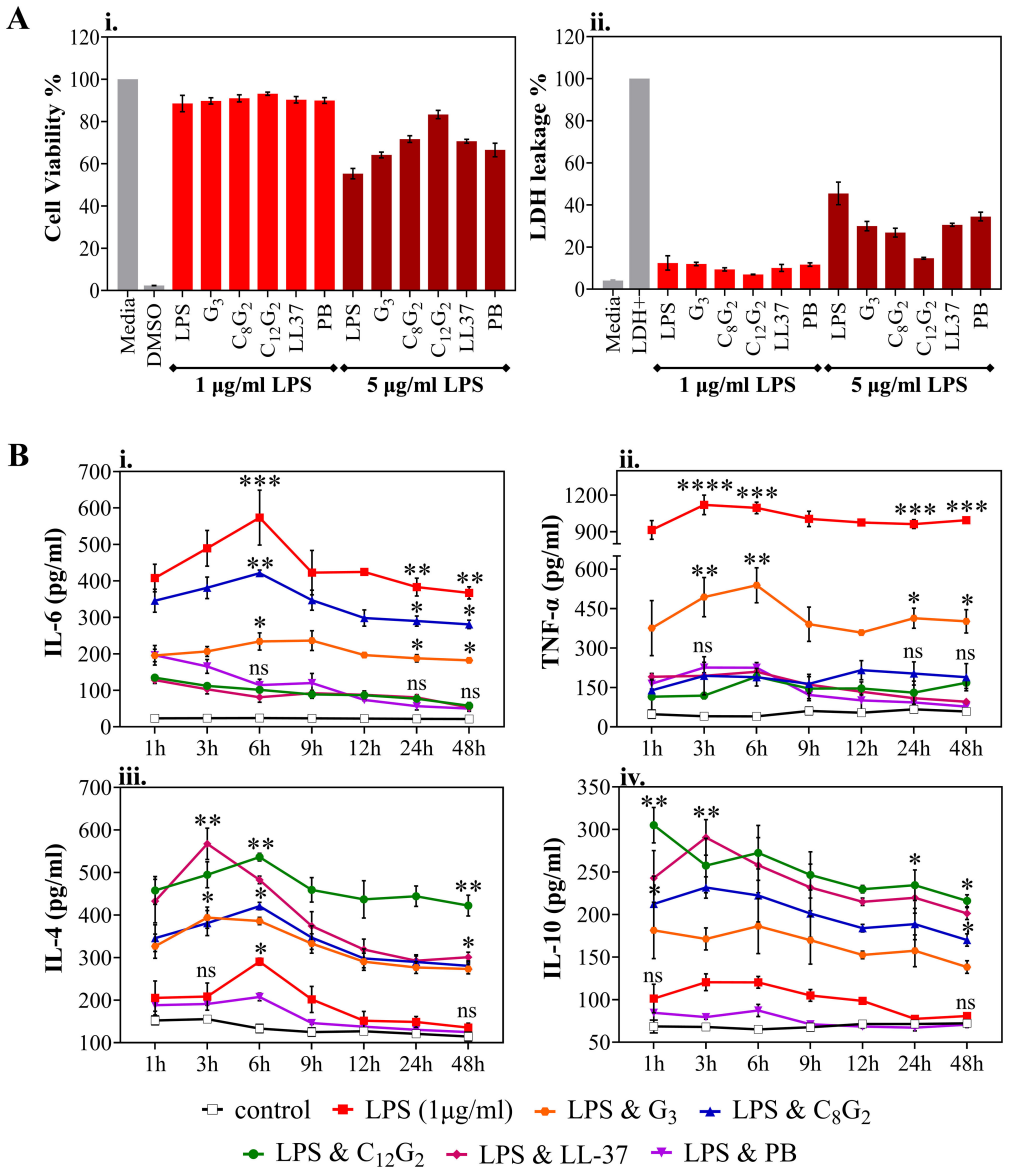
Effects of AMPs on viability and cytokine responses of LPS-stimulated THP-1 macrophages. **(A)** Cell viability and cytotoxicity following 24 h stimulation. **(i)** Cell viability (%) measured by MTT assay and **(ii)** LDH release (%) indicating membrane integrity in macrophage-like THP-1 cells treated with 1 or 5 μg/mL LPS in the presence or absence of AMPs (G_3_, C_8_G_2_, C_12_G_2_, LL-37) or PB. **(B)** ELISA quantification of cytokine secretion following co-stimulation with 1 μg/mL LPS and AMPs at their MIC concentrations over 1–48 h: **(i)** IL-6, **(ii)** TNF-α, **(iii)** IL-4, and **(iv)** IL10 (pg/mL). Polymyxin B was used as a positive control for LPS neutralization. Data are presented as mean ± SD (n = 3). Statistical analysis was performed using one-way ANOVA with Dunnett’s posthoc test for panel **(A)** and Tukey’s *post-hoc* test for panel **(B)** (*p < 0.05; **p < 0.01; ***p < 0.001; ****p < 0.0001; ns, not significant).

To assess the immunomodulatory effects of antimicrobial peptides, THP-1-derived macrophages were co-treated with LPS and the peptides at their MIC concentrations. Co-incubation of peptides with LPS resulted in modest improvements in cell viability and reduced LDH release compared with LPS treatment alone, particularly at 24 h (**Figure 3A**). Among these peptides evaluated C_12_G_2_ showed the greatest protective effect on cell viability.

LPS stimulation induced robust secretion of TNF-α and IL-6, whereas co-treatment with antimicrobial peptides significantly reduced the levels of these pro-inflammatory cytokines (**Figure 3B**). C_12_G_2_ and LL-37 produced the most pronounced suppression of TNF-α and IL-6. PB also reduced cytokine levels but was associated with greater cytotoxicity. In contrast, anti-inflammatory cytokines IL-4 and IL-10 were increased following peptide co-treatment, with C_12_G_2_ and LL-37 inducing the highest levels over the 48 h time course. Peptides alone did not induce significant cytokine release in unstimulated macrophages (**Supplementary Figure 7A**). These results demonstrate that antimicrobial peptides attenuate LPS-induced pro-inflammatory cytokine production while promoting anti-inflammatory cytokine responses.

### 3.5 AMPs promote M2-like surface marker expressions in LPS-stimulated macrophages

**Figure 4:**
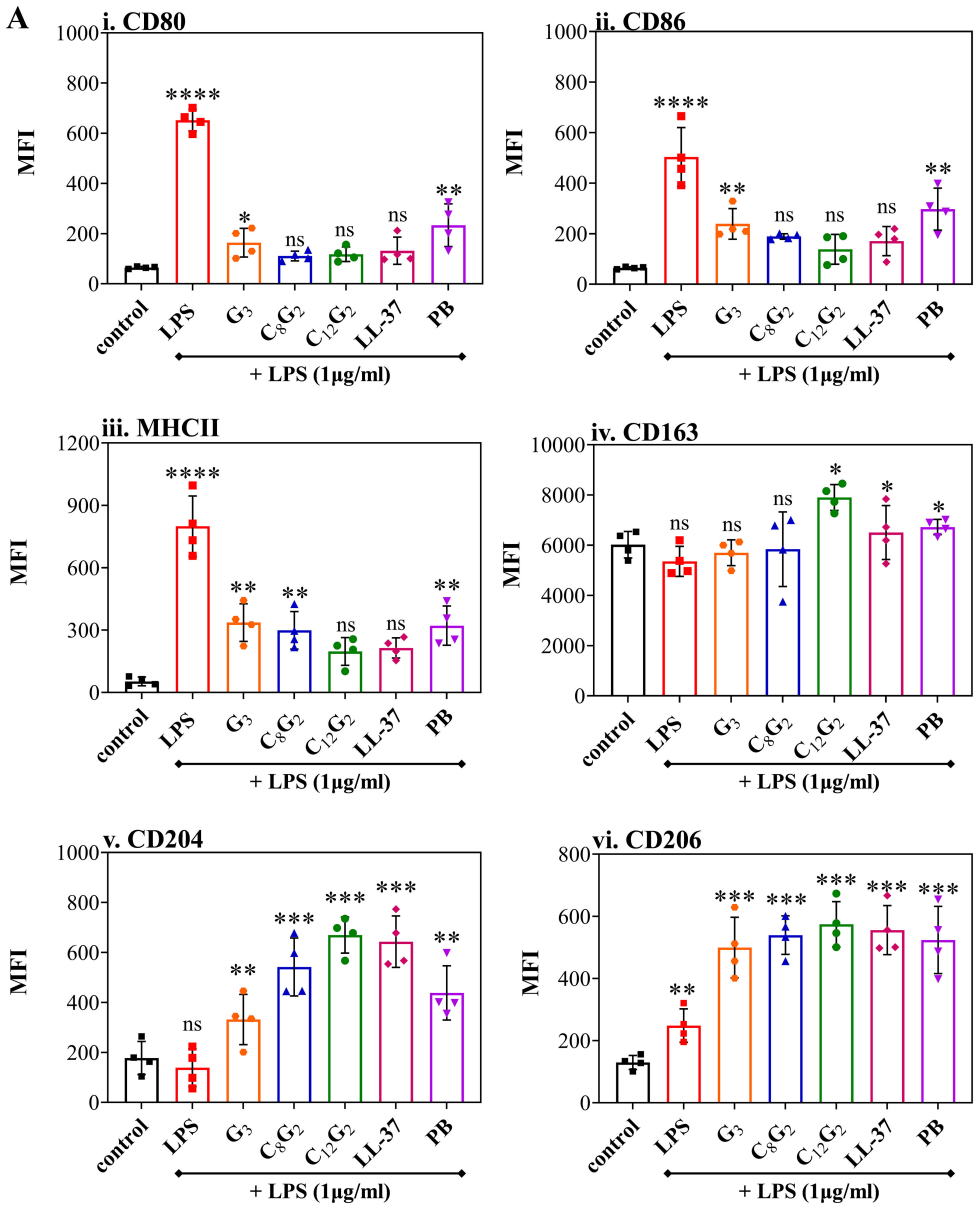
AMPs promote M2-like surface marker expression in LPS-stimulated THP-1 macrophages. **(A)** Flow cytometric analysis of macrophage surface markers following 24 h co-treatment with 1 µg/mL LPS and AMPs (G_3_, C_8_G_2_, C_12_G_2_, LL-37, or PB) at their MIC concentrations. Mean fluorescence intensity (MFI) values were normalized to unstimulated controls for **(i)** CD80, **(ii)** CD86, **(iii)** MHC class II, (M1-associated markers), and **(iv)** CD163, **(v)** CD204, **(vi)** CD206 (M2associated markers). Polymyxin B served as a positive control for LPS neutralization. Data are presented as mean ± SD (n = 3). Statistical significance was determined by one-way ANOVA with Tukey’s *post-hoc* test (*p < 0.05; **p < 0.01; ***p < 0.001; ****p < 0.0001; ns, not significant).

The effects of antimicrobial peptides on macrophage surface marker expression were examined by flow cytometry. LPS stimulation increased the expression of M1-associated surface markers CD80, CD86, and MHC class II. Co-treatment with antimicrobial peptides significantly reduced the expression of these markers (**Figure 4A**; panels i-iii).

Conversely, the M2-associated surface markers CD163, CD204, CD206 were upregulated in macrophages treated with peptides in the presence of LPS (**Figure 4A**; panels iv-vi). This effect was most pronounced for C_12_G_2_ and LL-37. PB showed weaker induction of M2 markers than the other peptides. These findings indicate that antimicrobial peptides shift the surface phenotype of LPSstimulated macrophages toward an M2-like profile.

### 3.6 AMPs reprogram inflammatory and polarization-associated gene expressions

**Figure 5:**
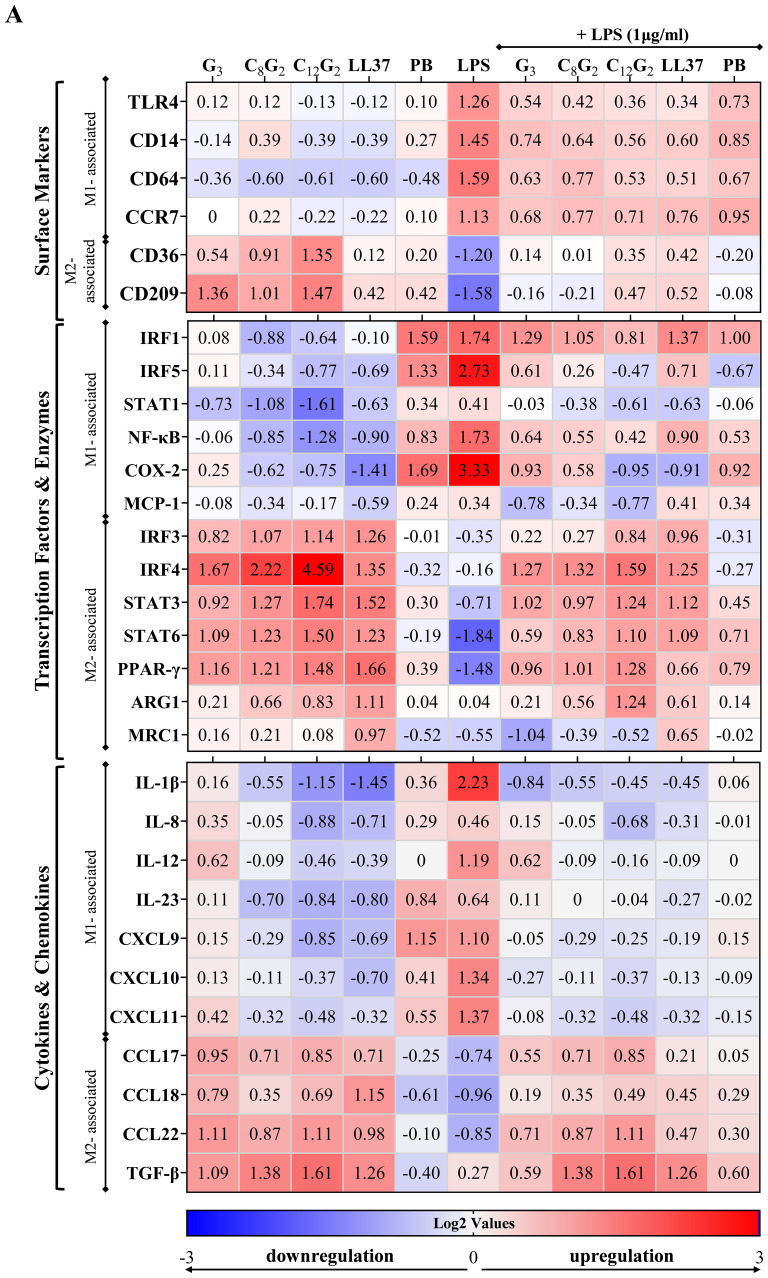
AMPs reprogram macrophage gene expression under LPS stimulation. **(A)** RT-qPCR heatmaps showing log_2_ fold changes in M1- and M2-associated gene expression following 24 h stimulation with 1 μg/mL LPS and/or AMPs (G_3_, C_8_G_2_, C_12_G_2_, LL-37, and PB). Surface markers, transcription factors, and effector molecules, cytokines, and chemokines. Blue indicates downregulation and red indicates upregulation (range: –3.00 to +3.00). Data are presented as mean ± SD (n = 3). Statistical significance was performed using one-way ANOVA with Tukey’s *post-hoc* test (*p < 0.05; **p < 0.01; ***p < 0.001; ****p < 0.0001; ns, not significant).

To investigate transcriptional changes underlying peptide-mediated macrophage reprogramming, RTqPCR analysis was performed on LPS- and peptide-treated macrophages. LPS stimulation increased the expressions of M1-associated genes, including *TLR4, CD14, CD64, CCR7, IL-1β, IL-12*, and *CXCL9-11* (**Figure 5**). Co-treatment with antimicrobial peptides reduced the expressions of these inflammatory markers, with synthetic peptides often showing greater effects than LL-37.

In parallel, antimicrobial peptides enhanced the expressions of genes associated with M2 polarization and immune regulation, including *IRF3, IRF4, STAT3, STAT6*, and *PPAR-γ.* Expressions of proinflammatory transcription factors *IRF1, IRF5, NF-κB*, and *STAT1* was reduced following peptide treatment. These transcriptional changes are consistent with a shift from an LPS-induced proinflammatory program toward an anti-inflammatory and reparative macrophage phenotype.

## 4 Discussion

AMPs are increasingly recognized not only for their antimicrobial activity but also for their capacity to modulate host immune responses, including inflammatory signaling, macrophage activation states, and broader innate immune reprogramming processes (16–20, 30). In this study, we demonstrate that rationally designed AMPs actively reprogram macrophage inflammatory responses and polarization states in the context of endotoxin-driven inflammation. Using a well-validated THP1 macrophage model, we show that synthetic AMPs suppress LPS-induced pro-inflammatory signaling, promote anti-inflammatory cytokine production, and shift macrophage phenotype toward an M2-like state, with low cytotoxicity and clear structure-function relationships (28).

A critical first step in this work was the establishment and validation of a robust macrophage polarization system. PMA-differentiated THP-1 cells displayed hallmark features of macrophage differentiation, including increased adherence, upregulation of CD11b and CD14, and preserved viability (44–46, 48–50). Functional differentiation was further confirmed through polarization studies under M0, M1 (classical-type), and M2 (alternative-type) conditions, supported by cytokine secretion profiles, surface marker expressions, and transcriptional analyses. The observed cytokine responses (e.g. TNF-α and IL-6 in M1; IL-10 and IL-4 in M2), together with polarization specific marker expressions, confirm that the differentiated THP-1 cells exhibit expected macrophage behavior. Moreover, their robust responsiveness to LPS stimulations and subsequent modulations by peptide treatment demonstrate intact macrophage signaling pathways. At the transcriptional level, the induction of canonical M1-associated genes (*IL-1β, CXCL9-11, IRF1, IRF5, STAT1*) and M2associated programs (*IL-10, TGF-β, IRF3, IRF4, STAT3, STAT6, PPAR-γ*) is consistent with established macrophage polarization paradigms and supports the use of this system for systematic immunomodulation studies and for investigating contemporary concepts of macrophage plasticity beyond the classical M1/M2 framework (27, 30–33, 35, 38, 39, 51, 52).

LPS stimulation induced a strong and sustained inflammatory response characterized by elevated TNF-α and IL-6 secretion and increased expression of M1-associated surface markers. Co-treatment with AMPs markedly attenuated these pro-inflammatory responses while simultaneously enhancing the production of anti-inflammatory cytokines such as IL-4 and IL-10. Among these peptides tested, the lipopeptide C_12_G_2_ and the natural AMP LL-37 consistently produced the most pronounced immunomodulatory effects, whereas PB, despite its LPS-neutralizing capacity, exhibited greater cytotoxicity and weaker induction of anti-inflammatory markers (14, 25). These findings underscore the advantage of rational peptide design over conventional antibiotics for achieving balanced immune regulation.

Notably, the immunomodulatory activity observed in this study is closely linked to the structural and physicochemical properties of peptides (3, 14, 53). G_3_, C_8_G_2_, and C_12_G_2_ differ in amphiphilic architecture, charge distribution, and hydrophobic modification, all of which govern their interactions with lipid membranes and LPS (5, 53). G_3_, containing three repeats of the IIKK motif, has a higher net positive charge that promotes electrostatic interactions with negatively charged bacterial membranes and endotoxins. However, its relatively low hydrophobicity limits membrane insertion, which is consistent with moderate immunomodulatory activity (3, 53).

In contrast, C_8_G_2_ and C_12_G_2_ incorporate lipid chains that enhance hydrophobic interactions and facilitate membrane insertion (7, 14, 53). The longer C12 chain in C_12_G_2_ further strengthens these interactions, likely improving its association with LPS and cellular membranes. This structural feature correlates with its enhanced ability to suppress pro-inflammatory cytokines and promote antiinflammatory responses. However, increased hydrophobicity also contributes to slightly elevated cytotoxicity at higher concentrations, highlighting the importance of balancing membrane activity with cellular compatibility (7, 14, 53). Together, these findings support the concept that an optimal balance between cationic charge and hydrophobicity governs AMP-mediated immunomodulation (5, 53).

It should be noted that peptides in this present study were evaluated at their respective MIC values, which introduced differences in absolute molar concentration between treatment groups. Therefore, some of the enhanced immunomodulatory activity observed for C_12_G_2_ may partially reflect concentration-dependent effects in addition to intrinsic structural advantages. However, the consistent activity of C_12_G_2_ across multiple readouts is also aligned with its physicochemical features, particularly the C12 hydrophobic tail, which is expected to strengthen membrane association and LPS interactions. Future studies using matched equimolar concentrations will be important to more clearly distinguish concentration-dependent from structure-dependent contributions.

Although direct LPS-binding assays were not performed, the functional data are consistent with a contribution of LPS neutralization. The ability of C_8_G_2_ and C_12_G_2_ to suppress LPS-induced TNF-α and IL-6 production compared to G_3_ suggests enhanced interaction with endotoxin. This is consistent with established mechanisms whereby cationic AMPs bind LPS through electrostatic interactions with negatively charged regions, combined with hydrophobic insertion into lipid A domains (5, 21, 25). These interactions are likely to reduce TLR4-mediated signaling, thereby attenuating downstream inflammatory responses (19, 21, 25). While future studies incorporating direct binding assays will further validate this mechanism, the current findings strongly support LPS neutralization as a key contributor to the observed immunomodulatory effects.

Compared to natural AMPs such as LL-37, the synthetic peptides investigated in this study offer several translational advantages (5, 6, 10). Natural AMPs are often limited by high production costs, and potential cytotoxic or off-target effects at higher concentrations (5, 6). In contrast, the short, repetitive sequences of G_3_, C_8_G_2_, and C_12_G_2_ enable cost-effective synthesis and facilitate rational structural modifications. For example, G_3_ demonstrates low cytotoxicity with preserved immunomodulatory activity, while C_8_G_2_ and C_12_G_2_ allow tuning of membrane interactions through lipid chain incorporation (3, 10). This modular design strategy enables optimization of efficacy and safety, addressing key limitations associated with natural peptides.

Additional considerations for clinical translation are susceptibility to proteolytic degradation and peptide stability under physiological conditions. G_3_ is significantly more stable than LL-37 under *in vitro* assessments against MMP-2 and MMP-9, the two enzymes that are hallmarks of chronic wound exudates (54). Many natural AMPs can also be rapidly inactivated by other serum proteases, ionic strength, and nonspecific binding to plasma proteins, which can limit *in vivo* efficacy. In contrast, the simplified short-sequence architectures of G_3_, C_8_G_2_, and C_12_G_2_ may provide advantages in manufacturability and opportunities for stability optimization through rational design. Furthermore, lipid modification in C_8_G_2_ and C_12_G_2_ may enhance peptide self-association, membrane affinity, and resistance to enzymatic degradation, as reported for other lipopeptide systems. Nevertheless, the stability of these peptides under biologically relevant conditions was not directly assessed in the present study. Future work should therefore examine serum stability, protease susceptibility, salt tolerance, and pharmacokinetic behavior to determine whether the *in vitro* immunomodulatory activity observed here is maintained *in vivo*, particularly given the increasing interest in engineering peptide platforms with improved pharmacological performance (17, 18).

Beyond these advantages, the synthetic peptides also exhibit distinct immunomodulatory profiles compared to LL-37. While LL-37 is a potent endogenous immunomodulator, its activity is constrained by its fixed structure and sequence, despite its well-established ability to modulate inflammatory signaling and neutralize LPS (19, 20, 25). In contrast, the designed peptides provide a tunable platform in which structural features can be systematically modified to regulate biological responses. G_3_ exhibits low cytotoxicity and moderate immunomodulation, whereas C_12_G_2_ shows enhanced suppression of pro-inflammatory cytokines and stronger promotion of anti-inflammatory pathways, likely due to improved LPS and membrane interactions. C_8_G_2_ displays intermediate behavior, further reinforcing the role of controlled amphiphilicity in determining function (23, 37).

At the cellular level, AMPs not only modulated cytokine production but also induced a clear shift in macrophage phenotype. LPS-driven upregulation of M1-associated markers CD80, CD86, and MHC class II was suppressed by peptide treatment, while M2-associated scavenger receptors CD163, CD204, and CD206 were enhanced. These changes reflect functional reprogramming of macrophages towards roles associated with resolution of inflammation, debris clearance, and tissue repair. For example, CD204 and CD206 are implicated in apoptotic cell clearance and extracellular matrix remodeling, processes essential for restoring tissue homeostasis following injury or infection.

Transcriptional profiling provided mechanistic insight into how synthetic AMPs mediate macrophage reprogramming. Peptide treatment reduced expressions of inflammatory signaling mediators downstream of TLR activation, including *TLR4, CD14, NF-κB, IRF1*, and *IRF5*, while enhancing expressions of transcription factors associated with anti-inflammatory and reparative programs, notably *IRF3, IRF4, STAT3, STAT6*, and *PPAR-γ.* The induction of *PPAR-γ* is particularly noteworthy, as this nuclear receptor plays a leading role in suppressing inflammatory gene expression while promoting lipid metabolism, and alternative macrophage activation. These findings align with current concepts of macrophage immunometabolism, in which metabolic pathways are tightly coupled to inflammatory phenotype and functional reprogramming (30–32). Upregulation of *MRC1* and *Arg1* in peptide-treated macrophages further supports engagement of *PPAR-γ* dependent pathways and reinforces the conclusion that AMPs actively drive macrophages toward an immunoregulatory phenotype rather than simply dampening inflammation (38, 39, 52).

**Figure 6:**
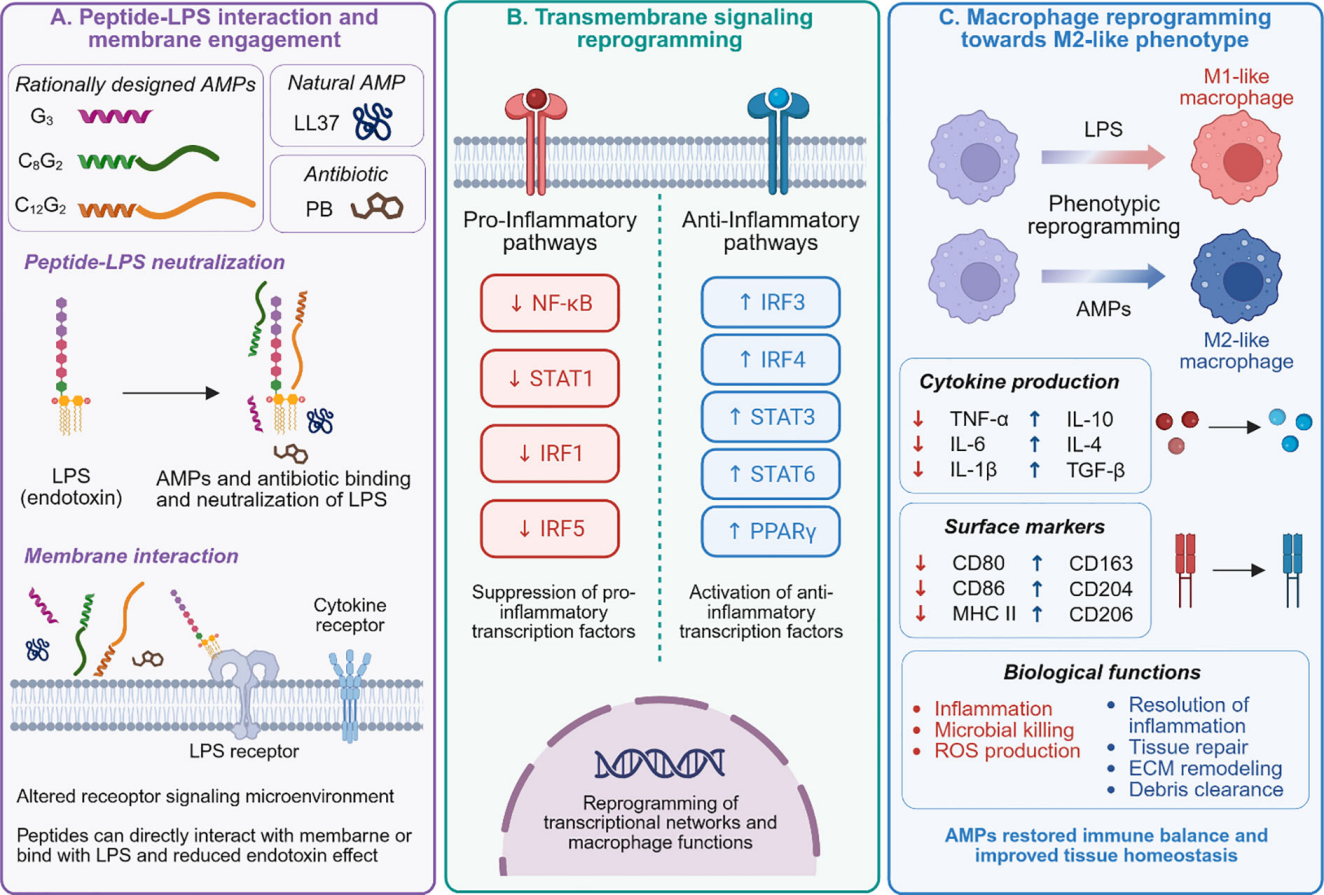
**(A)** Rationally designed, natural AMPs and antibiotics interact with LPS and cellular membranes, resulting in endotoxin neutralization and altered receptor signaling. **(B)** These interactions suppress pro-inflammatory pathways while activating anti-inflammatory regulators, leading to transcriptional reprogramming of macrophage function. **(C)** Downstream effects include reduced pro-inflammatory cytokine production, increased anti-inflammatory mediators, modulation of surface marker expression, and phenotypic polarization toward an M2-like macrophage state. This model summarizes the cytokine, gene expression, and flow cytometry findings observed in THP-1 macrophages following peptide treatment.

The integrated immunomodulatory effects of the designed AMPs are summarized schematically in **Figure 6**. This model illustrates how peptide-membrane interactions and LPS neutralization converge with intracellular signaling reprogramming to reshape macrophage phenotypes. By attenuating TLRdriven pro-inflammatory pathways while promoting *IRF3/IRF4*-, *STAT3/STAT6*-, and *PPAR-γ*associated transcriptional programs, the peptides drive a coordinated shift from an inflammatory M1like state toward a reparative M2-like phenotype.

The amphiphilic nature of the designed AMPs enables controlled membrane interaction and LPS engagement without inducing excessive host cell damage, distinguishing them from PB and highlighting the importance of balanced peptide design (3, 7, 14, 55).

A notable strength of this study is the demonstration that synthetic AMPs act as dual-function agents, coupling antimicrobial defense with immune reprogramming. Rather than functioning solely as LPS scavengers, these peptides reshape intracellular signaling networks that govern macrophage fate and may be relevant to emerging strategies aimed at therapeutic innate immune reprogramming (30–32). This distinction is critical, as excessive suppression of inflammation can be as detrimental as uncontrolled activation. By promoting M2-like polarization while preserving cell viability, the designed peptides achieve a more nuanced modulation of immune responses that may be advantageous in settings such as wound healing, sepsis-associated inflammation, and biomaterialassociated immune reactions.

Several limitations should be acknowledged. All experiments were performed using a THP-1-derived macrophage model, which, although highly reproducible and well characterized, cannot fully recapitulate the heterogeneity of primary macrophages or tissue-resident populations. Future studies using primary macrophages and *in vivo* models of endotoxemia or tissue injury will be essential to validate the translational relevance of these findings. Additionally, while transcriptional signatures strongly support engagement of specific signaling pathways, direct perturbation studies will be required to delineate causal relationships between peptide structure, receptor engagement, and downstream signaling.

In summary, this work demonstrates that rationally designed antimicrobial peptides can be engineered to exert precise immunomodulatory effects on macrophages, suppressing inflammatory activation while promoting reparative phenotypes. By integrating peptide biophysics with cellular immunology, our findings establish a framework for the development of next-generation immuneactive biomaterials that address infection and inflammation simultaneously. These insights expand the functional scope of AMPs beyond antimicrobial activity and highlight their potential as versatile tools for immune engineering and therapeutic intervention.

## 5 Conclusion

This study demonstrates that rationally designed AMPs can actively modulate macrophage polarization and inflammatory responses beyond their direct antimicrobial activity. Using a THP-1derived macrophage model, we show that the synthetic peptides, G_3_, C_8_G_2_, and C_12_G_2_ suppress LPSinduced pro-inflammatory cytokine production while promoting anti-inflammatory mediators and M2-associated surface marker expressions. At the transcriptional level, these peptides reprogram macrophage signaling pathways by downregulating pro-inflammatory regulators and enhancing *IRF3/IRF4*-, *STAT3/STAT6*-, and *PPAR-γ*- associated programs linked to immune resolution and tissue repair.

Importantly, the immunomodulatory effects observed here are closely linked to peptide structure and physicochemical design. By tuning amphiphilicity and hydrophobicity, the designed peptides achieve controlled membrane interactions, low cytotoxicity, and effective immune reprogramming, distinguishing them from conventional antibiotics such as PB. These findings highlight rational peptide design as a powerful strategy for balancing antimicrobial efficacy with immune compatibility.

Collectively, this work establishes synthetic antimicrobial peptides as a new class of immune-active biomaterials capable of integrating pathogen control with host immune modulation. By bridging peptide biophysics and macrophage immunology, our study provides framework for the development of next-generation therapeutics aimed at controlling infection-associated inflammation and supporting tissue repair.

## Data Availability

The original contributions presented in the study are included in the article/**Supplementary Material**. Further inquiries can be directed to the corresponding author.
