# Supplementary material for: Immunomodulatory effects of synthetic antimicrobial peptides on LPS-induced inflammatory responses in THP-1 macrophages
Source: Front Immunol. 2026 Jun 10;17:1829304. doi: 10.3389/fimmu.2026.1829304 (PMC13290583; doi:10.3389/fimmu.2026.1829304)
Supplement: Supplementary file 1 [file Supplementaryfile1.pdf]

Supplementary Material

Immunomodulatory Effects of Synthetic Antimicrobial Peptides on LPS-Induced Inflammatory Responses in THP-1 Macrophages

Ilayda Akbulut[1], Ziyun Zhang[2], Tracy Hussell[2], Jeremy P. Derrick[2], Jian Ren Lu[1*]

[1]Biological Physics Laboratory, Department of Physics and Astronomy, School of Natural Sciences, Faculty of Science and Engineering, The University of Manchester, Oxford Road, Manchester M13 9PL, UK.

[2]Division of Infection, Immunity & Respiratory Medicine, School of Biological Sciences, Faculty of Biology, Medicine and Health, The University of Manchester, Oxford Road, Manchester M13 9PL, UK

*To whom all correspondence should be addressed, Email: j.lu@manchester.ac.uk

Keywords:

antimicrobial peptides, inflammatory kinetics, innate immune modulation, lipopolysaccharide neutralization, macrophage polarization, THP-1-derivedmacrophages

1 Supplementary Figures

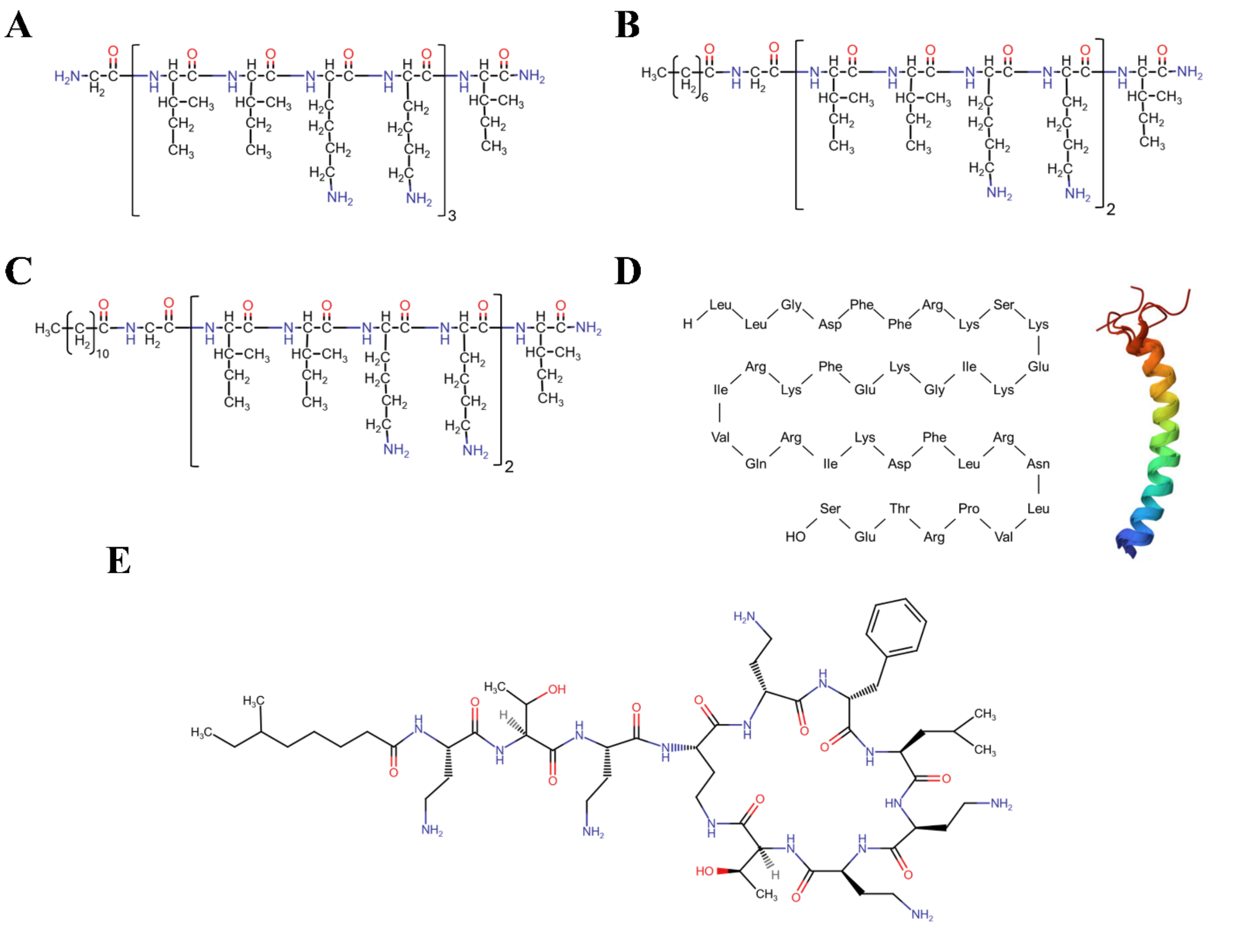

Supplementary Figure S1. Chemical structures of the antimicrobial peptides used in this study. **(A)** G_3, **(B)** C_8G_2, **(C)** $C_{12}G_2$, **(D)** LL-37 (amino acid sequence corresponding to Protein Data Bank entry 2K6O), and **(E)** polymyxin B.

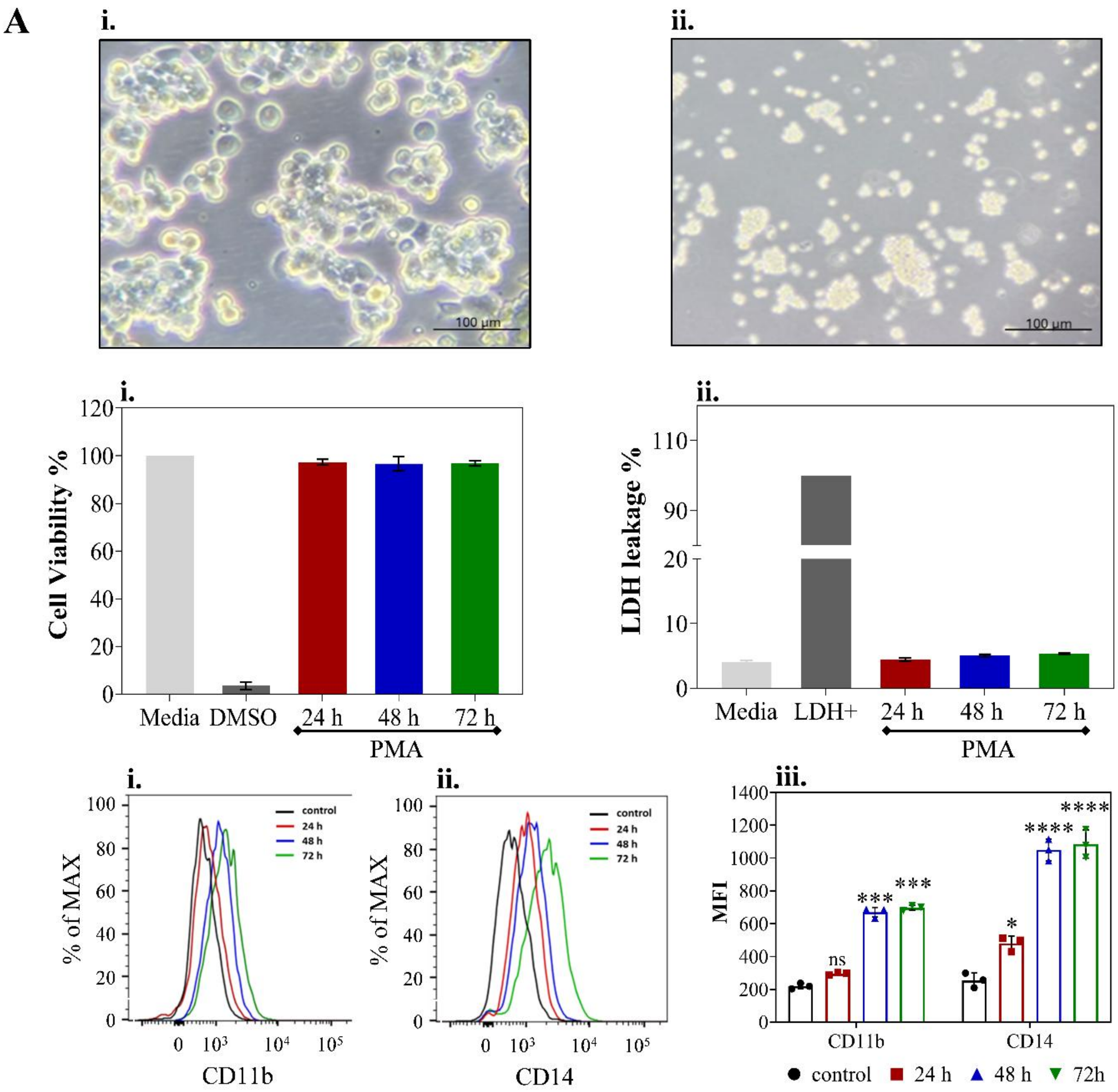

Supplementary Figure S2. PMA-induced differentiation of THP-1 monocytes into macrophage-like cells. **(A)** Representative light microscopy images of **(i)** undifferentiated THP-1 monocytes and **(ii)** THP-1 cells differentiated into macrophage-like cells following 48 h treatment with 100 ng/mL PMA (×20 magnification; scale bar = 100 µm). **(B)** Cell viability and cytotoxicity following PMA treatment: **(i)** cell viability (%) assessed by MTT assay and **(ii)** LDH release (%) in THP-1 cells treated with 100 ng/mL PMA for 24, 48, and 72 h. **(C)** Flow cytometric analysis of macrophage differentiation markers following PMA treatment: **(i)** CD11b and **(ii)** CD14 expression profiles, and **(iii)** quantification of mean fluorescence intensity (MFI) for CD11b and CD14. Data are presented as mean ± SD (n = 3). Statistical analysis was performed using one-way ANOVA with Dunnett's post-hoc test (*$p < 0.05$; **$p < 0.01$; ***$p < 0.001$; ****$p < 0.0001$; ns, not significant).

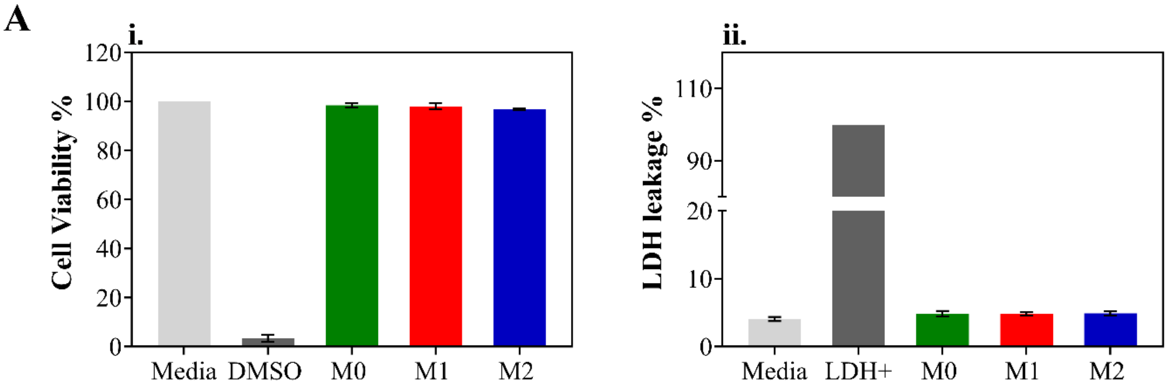

Supplementary Figure S3. Viability and cytotoxicity of polarized THP-1-derived macrophages. **(A)** Assessment of cell viability and membrane integrity following macrophage polarization: **(i)** cell viability (%) of macrophage-like THP-1 cells after 24 h stimulation with 20 ng/mL IFN-γ plus 1 μg/mL LPS (M1) or 20 ng/mL IL-4 (M2), determined by MTT assay, and **(ii)** LDH release (%) in differentiated and 24 h-polarized THP-1 macrophages. Data are presented as mean ± SD (n = 3). Statistical significance was determined by one-way ANOVA with Dunnett's post-hoc test (**p* < 0.05; ***p* < 0.01; ****p* < 0.001; ****p* < 0.0001; ns, not significant).

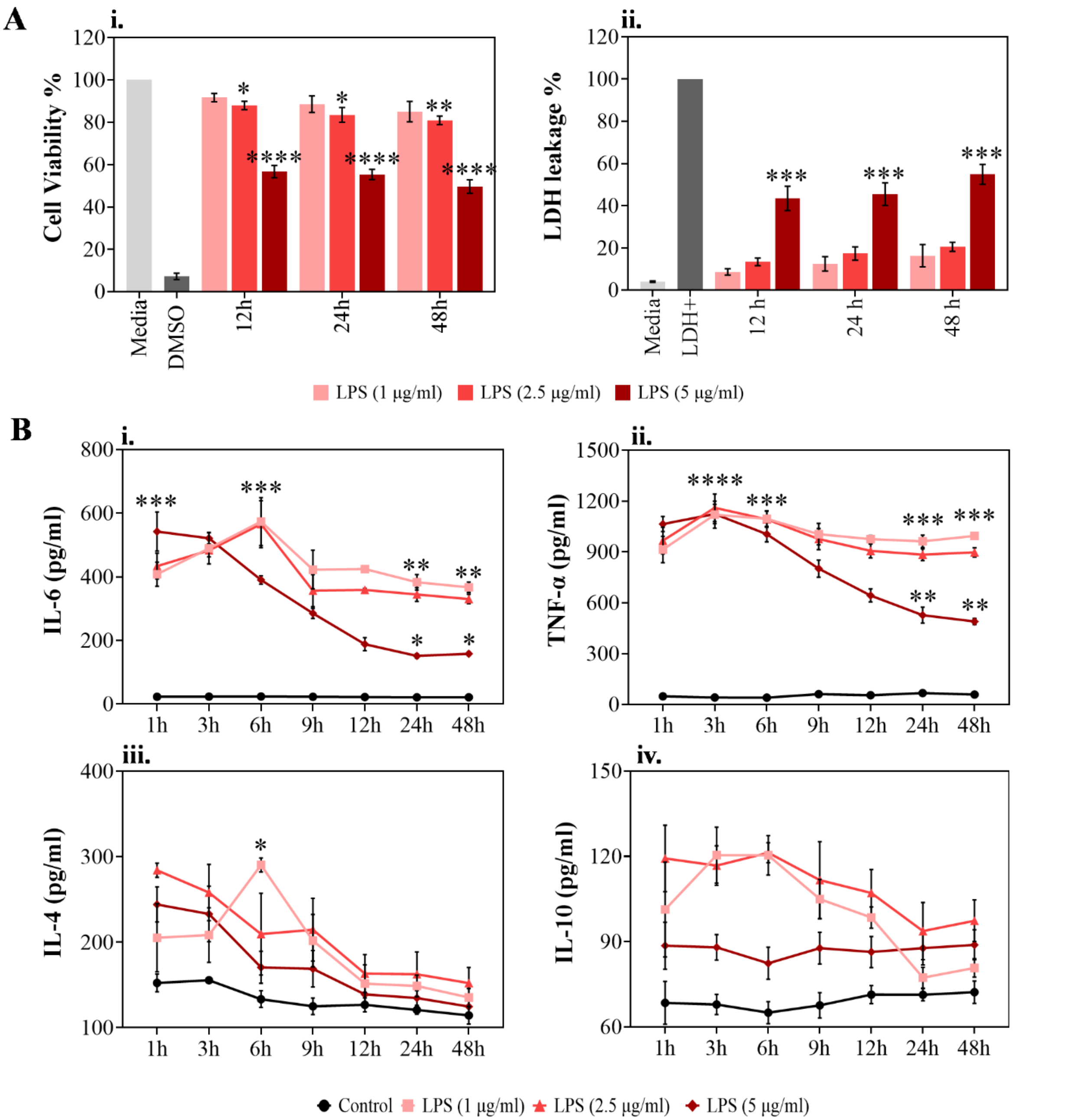

Supplementary Figure S4. Effects of LPS stimulation on differentiated THP-1-derived macrophages. **(A)** Cell viability and cytotoxicity following LPS treatment: **(i)** cell viability (%) of macrophage-like THP-1 cells after 12, 24, and 48 h exposure to LPS, assessed by MTT assay, and **(ii)** LDH release (%) under the same conditions. **(B)** Cytokine secretion profiles measured by enzyme-linked immunosorbent assay (ELISA) at 1–48 h following stimulation with increasing concentrations of LPS: **(i)** TNF-α, **(ii)** IL-6, **(iii)** IL-4, and **(iv)** IL-10 (pg/mL). Data are presented as mean ± SD (n = 3). Statistical analysis was performed using one-way ANOVA with Dunnett's post-hoc test for panel (A) and Tukey's post-hoc test for panel (B) (*$p < 0.05$; **$p < 0.01$; ***$p < 0.001$; ****$p < 0.0001$; ns, not significant).

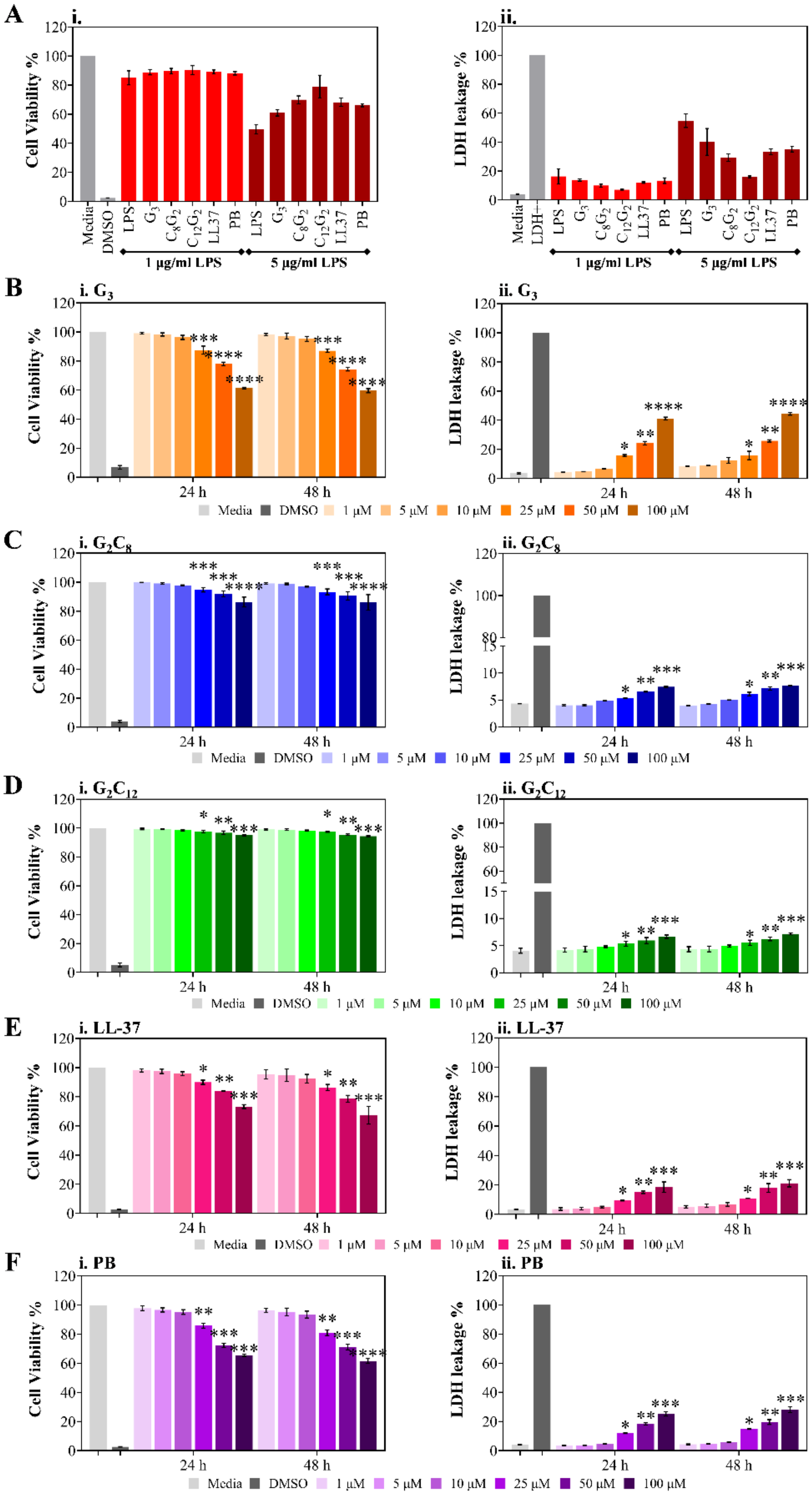
A
i.
ii.
Cell Viability %
LDH leakage %
Media
DMSO
LDH+
LPS
G3
C8G2
C12G2
LL37
PB
1 µg/ml LPS
5 µg/ml LPS
B
i. G3
ii. G3
C
i. G2C8
ii. G2C8
D
i. G2C12
ii. G2C12
E
i. LL-37
ii. LL-37
F
i. PB
ii. PB
24 h
48 h
Media
DMSO
1 µM
5 µM
10 µM
25 µM
50 µM
100 µM

Supplementary Figure S5. Viability and cytotoxicity of AMP- and LPS-stimulated THP-1 macrophages. **(A)** Cell viability (%) and LDH release (%) following 48 h co-treatment of macrophage-like THP-1 cells with AMPs and 1 µg/mL LPS: **(i)** MTT-based cell viability and **(ii)** LDH release. **(B-F)** Individual analyses of macrophage viability and cytotoxicity following 48 h treatment with **(B)** G_3, **(C)** C_8G_2, **(D)** $C_{12}G_2$, **(E)** LL-37, and **(F)** PB: **(i)** MTT assay results and **(ii)** LDH assay results. Data are presented as mean ± SD (n = 3). Statistical analysis was performed using one-way ANOVA with Dunnett's post-hoc test (*$p < 0.05$; **$p < 0.01$; ***$p < 0.001$; ****$p < 0.0001$; ns, not significant) .

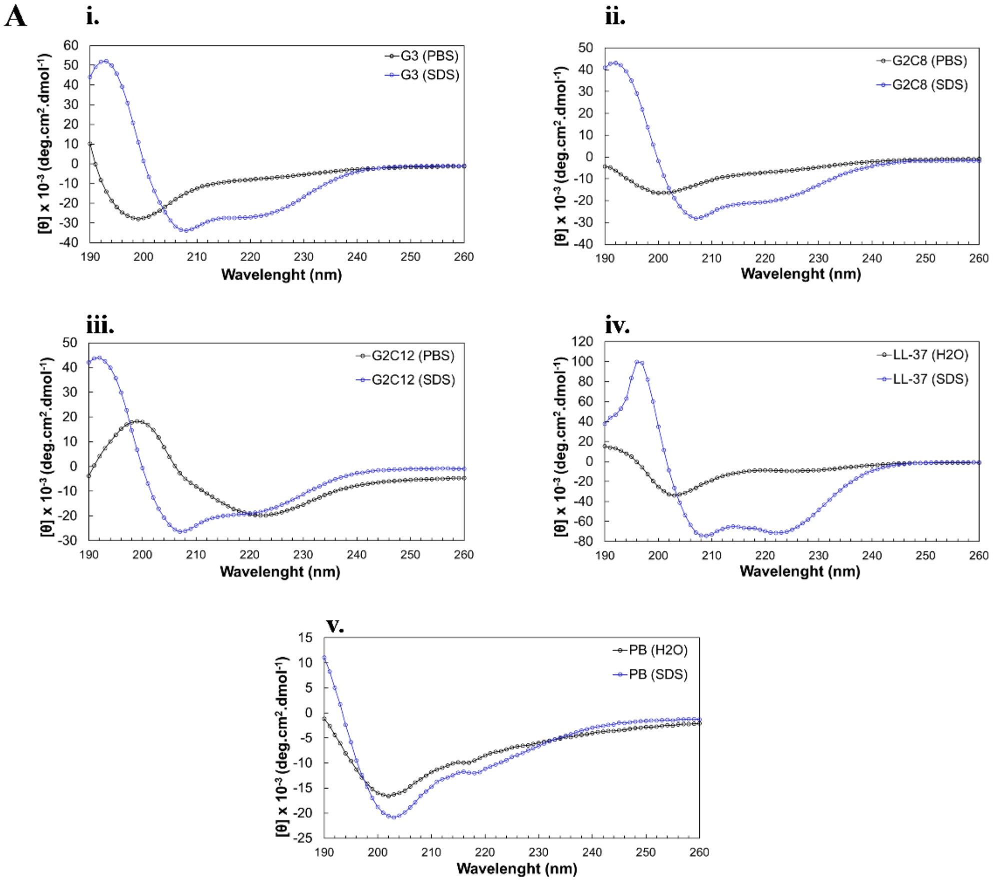

Supplementary Figure S6. Secondary structure analysis of AMPs by circular dichroism spectroscopy. **(A)** Circular dichroism spectra of **(i)** G_3, **(ii)** C_8G_2, **(iii)** $C_{12}G_2$, **(iv)** LL-37, and **(v)** PB measured in aqueous buffer (PBS or H_2O) and SDS micelles. Spectra illustrate environment-dependent secondary structure transitions in aqueous and membrane-mimicking conditions.

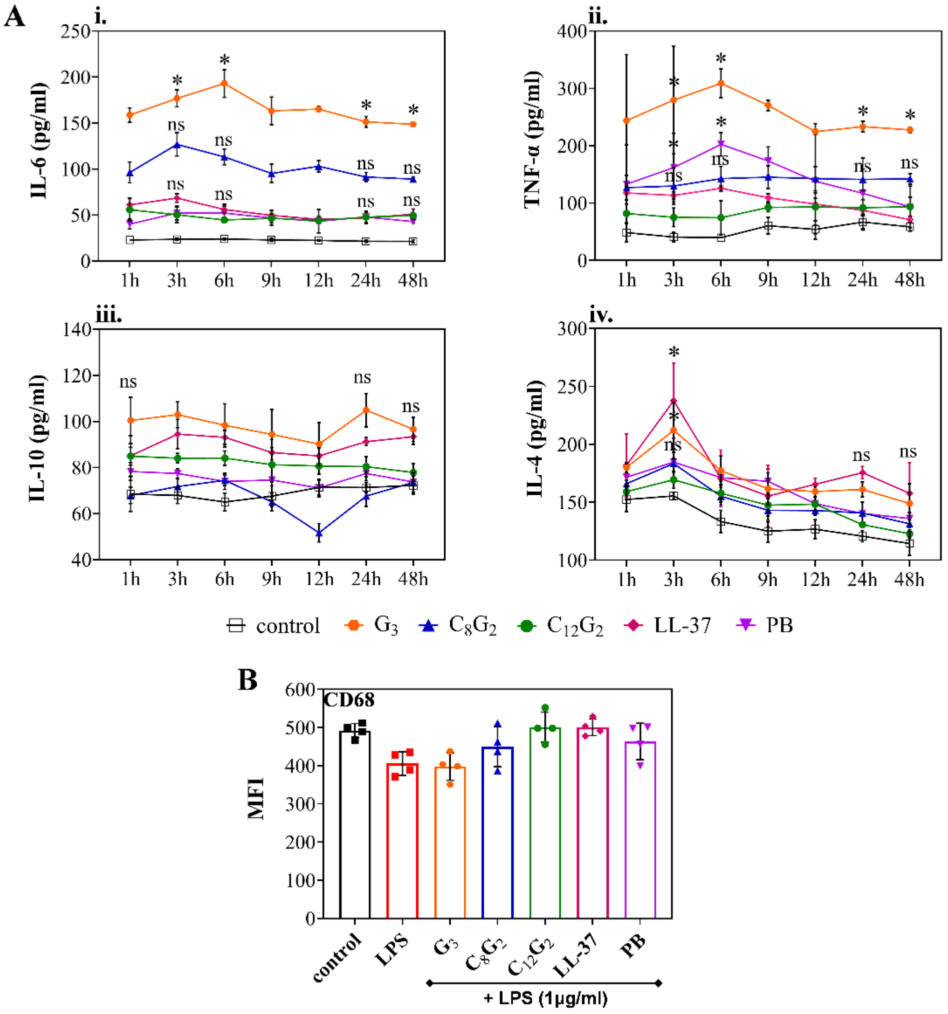

Supplementary Figure S7. Cytokine secretion and activation marker expression in peptide-stimulated THP-1 macrophages. **(A)** Cytokine secretion profiles measured by ELISA at 1–48 h following stimulation with AMPs at their minimum inhibitory concentration (MICs): **(i)** IL-6, **(ii)** TNF-α, **(iii)** IL-10, and **(iv)** IL-4 (pg/mL). **(B)** Flow cytometric analysis of macrophage activation marker CD68: **(i)** Mean fluorescence intensity (MFI) following treatment with LPS alone or in combination with AMPs. Data are presented as mean ± SD (n = 3). Statistical analysis was determined by one-way ANOVA with Tukey's post-hoc test (*$p < 0.05$; **$p < 0.01$; ***$p < 0.001$; ****$p < 0.0001$; ns, not significant).

2 Supplementary Table

Supplementary Table S1. Primer sequences used for RT-qPCR

Gene	Forward Primer (5' – 3')	Reverse Primer (5' – 3')
B2M	AAGGACTGGTCTTTCTATCTC	GATCCCACTTAACTATCTTGG
IL-1β	CTAAACAGATGAAGTGCTCC	GGTCATTCTCCTGGAAGG
IL-8	GTTTTTGAAGAGGGCTGAG	TTTGCTTGAAGTTTCACTGG
IL-12A	GCACAGTGGAGGCCTGTTTA	GCCAGGCAACTCCCATTAGT
IL-13	ATCACCCAGAACCAGAAG	ATGCAAGCTGGAAAACTG
IL-23A	AAAATCCTTCGCAGCCTCCA	ATCCTTGAGCTGCTGCCTTT
IFN-γ	TGCAATCTGAGCCAGTGCTT	GCACCAGGCATGAAATCTCC
CXCL9	ATTGGTGCCCAGTTAGCCTC	TTCTGGCCACAGACAACCTC
CXCL10	CTGAGCCTACAGCAGAGGAAC	GATGCAGGTACAGCGTACAGT
CXCL11	TTCCACTGCCCAAAGGAGTC	CTCCGATGGTAACCAGCCTT
CCL17	TCAGAGAGAAGTGACTTTGAGC	AGGGAGACACCCTGGTTTTG
CCL18	CTATACCTCCTGGCAGATTC	CTCTCTTGGTTAGGAGGATG
CCL22	GCCTACTCTGATGACCGTGG	AGAGAGTTGGCACAGGCTTC
CCL24	TCTGCAAGGACCCGAGCTAT	GATGATGTGGTGGGCACAGA
TGF-β1	CCCCTACATTTGGAGCCTGG	CACGTAGTACACGATGGGCA
IRF1	GACCCTGGCTAGAGATGCAG	TGCTTTGTATCGGCCTGTGT
IRF5	CTGGAAAGCGAGCTCGGAC	TCCCCGTTGACCCATTGAAG
STAT1	TCTGTGTCTGAAGTTCACCCTT	GCGAATTTGCTGGCCTTTCT

NF-κB1	GCTTAGGAGGGAGAGCCCAC	AACATTTGTTCAGGCCTTCCC
COX-2 (PTGS2)	GTTCCACCCGCAGTACAGAA	AGGGCTTCAGCATAAAGCGT
MCP-1 (CCL2)	CCCAAGCAGAAGTGGGTTCA	GTGTCTGGGGAAAGCTAGGG
IRF3	TTGACCTGGGGCCCTTCATT	CACACAGAACCAGAGGGCAT
IRF4	TGTCCCATGACGTTTGGACC	GTGGGGCACAAGCATAAAAGG
STAT3	GGTACATCATGGGCTTTATC	TTTGCTGCTTTCACTGAATC
STAT6	TCGCTGGACAGAGCTACAGA	AAGTCGACATAGAGCCGCTG
PPAR-γ	AGCAAACCCCTATTCCATGCT	TGGCATCTCTGTGTCAACCA
ARG1	TCTTCTGTGATGTAGAGACC	TACAAAACAGGGCTACTCTC
MRC1	GCCTCGTTGTTTTGCGTCTT	GAGAACAGCACCCGGAATGA
TLR2	CTTTCAACTGGTAGTTGTGG	GGAATGGAGTTTAAAGATCCTG
TLR4	GATTTATCCAGGTGTGAAATCC	TATTAAGGTAGAGAGGTGGC
CD14	GTACTCCCGCCTCAAGGAAC	AAAGTGCAAGTCCTGTGGCT
CD64	TGATGGGCAAGTGGTAGACAC	GATTCTGTAGCTGGGGGTCG
CCR7	AGGGGTAGTGCGAGGC	CCAGCACGCTTTTCATTGGTT
CD36	GGCAACAAACCACACACTGG	GTCCTACACTGCAGTCCTCA
CD209	GAGAGGCCTTGGATTCCGAC	AGAGCGTGAAGGAGAGGAGT

Supplementary Table S2. Physicochemical properties of AMPs and PB used in this study

Peptide	Amino Acid Number	Charge (pH 7.4)	Molecular Mass (g/mol)	Retention Time (min)	MIC (µM) *E. coli*
G_3	14	+7	1635.3	19.5	8 ± 2
C_8G_2	10	+4	1278.6	24.3	13 ± 4
$C_{12}G_2$	10	+4	1334.6	26.0	32 ± 12
LL-37	37	+6	4493.3	43.0	4 ± 2
Polymyxin B	10	+5	1301.6	10.5	4 ± 2

Supplementary Table S3. Secondary structures of AMPs and PB used in this study

	G_3	C_8G_2	$C_{12}G_2$	LL-37	PB
**PBS**	Min at 200 nm Random coiling	Min at 200 nm Random coiling	Min at 220 nm Max at 200 nm β-sheet structure	-	-
**H_2O**	-	-	-	Min at 202 nm Random coiling β-sheet structure	Min at 202 nm Random coiling
**SDS**	Min at 208, 222 nm Max at 193 nm α-helical structure	Min at 208, 222 nm Max at 193 nm α-helical structure	Min at 208, 222 nm Max at 193 nm α-helical structure	Min at 208, 222 nm Max at 193 nm α-helical structure	Min at 203, 220 nm α-helical structure

Supplementary Table S4. Fold change of gene expression signatures associated with M1 and M2 macrophage polarization in THP-1 derived macrophages

	M1	M2
TLR2	1.30	0.68
TLR4	3.34	0.36
CD14	1.99	0.41
CD64	1.23	0.50
CCR7	2.85	0.37
CD36	0.32	3.36
CD209	0.28	3.71
IRF1	4.23	0.34
IRF5	2.48	0.32
STAT1	3.10	0.28
NF-κB	3.81	0.77
COX-2	11.39	0.32
MCP-1	1.91	1.31
IRF3	0.51	4.23
IRF4	0.65	3.56
STAT3	0.53	4.53
STAT6	0.36	2.00
PPAR-γ	0.48	3.43
ARG1	0.35	0.93
MRC1	0.24	4.72
IL-1β	3.73	0.51
IL-8	1.92	1.47
IL-12	3.14	0.27
IL-13	1.67	0.82
IL-23	1.60	0.68
IFN-γ	12.13	0.28
CXCL9	2.41	0.48
CXCL10	2.79	0.40
CXCL11	1.95	0.49
CCL17	0.32	2.48
CCL18	0.41	1.69
CCL22	0.27	2.58
CCL24	0.36	2.48
TGF-β	1.09	3.94

Supplementary Table S5. Fold change of macrophage-associated gene expression changes following peptide treatment under basal and LPS-stimulated conditions

	G_3	C_8G_2	$C_{12}G_2$	LL37	PB	LPS	G_3	C_8G_2	$C_{12}G_2$	LL37	PB
**TLR4**	1.09	1.09	0.91	0.92	1.07	2.39	1.45	1.34	1.28	1.27	1.70
**CD14**	0.91	1.31	0.76	0.76	1.21	2.73	1.67	1.56	1.47	1.52	1.80
**CD64**	0.78	0.66	0.66	0.66	0.72	3.01	1.55	1.71	1.44	1.42	1.60
**CCR7**	1.00	1.16	0.86	0.86	1.07	2.19	1.60	1.71	1.64	1.69	1.90
**CD36**	1.45	1.88	2.55	1.09	1.15	0.44	1.10	1.01	1.27	1.34	0.90
**CD209**	2.57	2.01	2.77	1.34	1.34	0.33	0.90	0.86	1.39	1.43	0.90
**IRF1**	1.06	0.54	0.64	0.93	3.01	3.34	2.45	2.07	1.75	2.58	2.00
**IRF5**	1.08	0.79	0.59	0.62	2.51	6.63	1.53	1.20	0.72	1.64	0.60
**STAT1**	0.60	0.47	0.33	0.65	1.27	1.33	0.98	0.77	0.66	0.65	1.00
**NF-κB**	0.96	0.55	0.41	0.54	1.78	3.32	1.56	1.46	1.34	1.87	1.40
**COX-2**	1.19	0.65	0.59	0.38	3.23	10.06	1.91	1.49	0.52	0.53	1.90
**MCP-1**	0.95	0.79	0.89	0.66	1.18	1.27	0.58	0.79	0.59	1.33	1.30
**IRF3**	1.77	2.10	2.20	2.39	0.99	0.78	1.16	1.21	1.79	1.95	0.80
**IRF4**	3.18	4.66	24.08	2.55	0.80	0.90	2.41	2.50	3.01	2.38	0.80
**STAT3**	1.89	2.41	3.34	2.87	1.23	0.61	2.03	1.96	2.36	2.17	1.40
**STAT6**	2.13	2.35	2.83	2.35	0.88	0.28	1.51	1.78	2.14	2.13	1.60
**PPAR-γ**	2.23	2.31	2.79	3.16	1.31	0.36	1.95	2.01	2.43	1.58	1.70
**ARG1**	1.16	1.58	1.78	2.16	1.03	1.03	1.16	1.47	2.36	1.53	1.10
**MRC1**	1.12	1.16	1.06	1.96	0.70	0.68	0.49	0.76	0.70	1.57	1.00
**IL-1β**	1.12	0.68	0.45	0.37	1.28	4.69	0.56	0.68	0.73	0.73	1.00
**IL-8**	1.27	0.97	0.54	0.61	1.22	1.38	1.11	0.97	0.62	0.81	1.00
**IL-12**	1.54	0.94	0.73	0.76	1.00	2.28	1.54	0.94	0.90	0.94	1.00
**IL-23**	1.08	0.62	0.56	0.57	1.79	1.56	1.08	1.00	0.97	0.83	1.00
**CXCL9**	1.11	0.82	0.55	0.62	2.22	2.14	0.97	0.82	0.84	0.88	1.10
**CXCL10**	1.09	0.93	0.77	0.62	1.33	2.53	0.83	0.93	0.77	0.91	0.90
**CXCL11**	1.34	0.80	0.72	0.80	1.46	2.58	0.95	0.80	0.72	0.80	0.90
**CCL17**	1.93	1.64	1.80	1.64	0.84	0.60	1.46	1.64	1.80	1.16	1.00
**CCL18**	1.73	1.27	1.61	2.22	0.66	0.51	1.14	1.27	1.40	1.37	1.20
**CCL22**	2.16	1.83	2.16	1.97	0.93	0.55	1.64	1.83	2.16	1.39	1.20
**TGF-β**	2.13	2.60	3.05	2.39	0.76	1.21	1.51	2.60	3.05	2.39	1.50
